# Microcrystallization Effects in Borosilicate Bioactive Glasses: Controllable Release of Bioactive Elements and In Vitro Degradation Properties

**DOI:** 10.3390/ma17010032

**Published:** 2023-12-20

**Authors:** Chengyun Jin, Minhui Zhang, Jian Lin

**Affiliations:** 1 School of Materials Science and Engineering, Tongji University, Shanghai 201804, China; 2130608@tongji.edu.cn (C.J.); 1710808@tongji.edu.cn (M.Z.); 2 Key Laboratory of Advanced Civil Engineering Materials, Ministry of Education, Tongji University, Shanghai 200092, China

**Keywords:** borosilicate bioactive glass, microcrystallized glass, in vitro degradation, bioactive element

## Abstract

Borosilicate bioactive glasses exhibit excellent bioactivity and degradation properties; however, they suffer from the rapid release of bioactive elements at the initial stage of their degradation. Excessive local concentrations (such as those of B) can affect cell proliferation. Moreover, the degradation and mineralization ability of these glasses deteriorate at the later stages. Aiming to balance the release of bioactive elements during the whole process, herein, a borosilicate bioactive glass 18SiO_2_–6Na_2_O–8K_2_O–8MgO–22CaO–2P_2_O_5_–36B_2_O_3_ (mol%) was prepared using the melting method. Further, the effects of microcrystallization on the release of bioactive elements and in vitro degradation were studied. Results show that after heat treatment at temperatures over 620 °C, multiple microcrystalline phases, including Ca_2_SiO_4_, CaB_2_O_4_, and CaMgB_2_O_5_, form in the glass. The glass samples heat-treated within the range of 620–640 °C undergo appropriate devitrification degrees, decelerating the rate of pH increase of the immersion solution during the initial stage in comparison to those treated at lower temperatures. This results in a more continuous release of all bioactive elements and allows better control of the overall degradation. Contrarily, the more extensive devitrification degrees of glass samples heat-treated at higher temperatures reverse the pH increase and degradation trends. Since bone marrow mesenchymal stem cells and mouse embryonic osteoblast cells are pH-sensitive, inducing a suitable degree of devitrification proved to favor cell viability and enhance the mineralization capacity. Thus, different microcrystallization degrees provide new approaches for controlling the degradation and release of bioactive elements, resulting in the simultaneous enhancement of biosafety and bioactivity.

## 1. Introduction

In recent decades, fractures and bone defects caused by osteoporosis as well as other bone diseases in the elderly, which considerably reduce their living quality, have received increasing attention [1,2,3]. Borosilicate bioactive glasses have emerged as promising materials for bone repair. Compared with silicate bioactive glasses, they exhibit superior bioactivities and faster degradation rates owing to their lower Si content [4]. The skeleton structure of borosilicate bioactive glasses primarily comprises [BO_3_], [BO_4_], and [SiO_4_]. Accordingly, controlled biodegradation performance can be achieved by adjusting these components [5,6]. Borosilicate bioactive glasses can firmly bond with bones, support soft-tissue infiltration, and promote bone growth and osteoconductivity [7,8,9]. Releases of bioactive elements, such as Ca, P, B, Si, and Na, from borosilicate bioactive glasses can stimulate human bone-regeneration gene expression as well as promote cell proliferation and vascular fiber regeneration. In addition, these elements are necessary raw materials for the bone mineral phase, which enhances the regenerative capacity of bone tissues [7,10,11].

However, the degradation of borosilicate bioactive glasses is characterized by rapid degradation at the initial stage and slow degradation at the later stage, which easily leads to the local enrichment of multiple bioactive components at the beginning and weak bone-regeneration ability later on [5,12]. The moderate release of B has been shown to facilitate tissue infiltration and cell proliferation [13,14], whereas rapid increases in local concentrations are counterproductive [15,16]. Various methods are generally adopted to regulate the initial degradation rate, including component adjustment [5,6], novel preparation methods [17,18], morphology and particle size adjustment [19], and surface premineralization [20]. However, apart from improving the initial degradation, most of these methods also have an inhibitory effect on the later release of bioactive elements, which is not conducive to mineralization.

In principle, microcrystallization can be adopted to modulate the bioactivity of bioactive glasses. Microcrystallized glasses can be obtained via the heat treatment of precursor glasses [4,21]. Uniform and fine-grained microcrystals can grow by controlling the heat-treatment parameters such as temperature, time, and rate [22]. The crystalline phase is often used as a toughening phase in such glasses, endowing them with superior mechanical properties and chemical stability compared to the original glasses [10,21,23]. For biomedical applications, a portion of the crystalline phase can be converted to amorphous calcium phosphate in vivo and in vitro [24,25], promoting the formation of bone mineral phases. Several studies [26,27,28] have shown that microcrystallized borosilicate bioactive glasses can effectively slow the release of BO_3_^3−^ and have good mineralization properties and excellent osteogenic activity. However, most of these studies focused on the excellent mineralization properties resulting from structural changes. In contrast, the effects of microcrystallization on the release of bioactive elements and the modulation of the overall biodegradation have rarely been reported.

Herein, with the aim of regulating the degradation of borosilicate bioactive glass, the effects of microcrystallization on the release of bioactive elements, and the in vitro degradation mechanism were investigated. By optimizing the heat treatment for microcrystallization, the relationships among preparation, microcrystalline precipitation, bioactive ingredient release, and in vitro degradation and mineralization were studied to identify a balance between the initial sudden release and the continuous decrease of ingredient release in the later stage, aiming to ensure biosafety and equilibrium during the whole degradation process.

## 2. Materials and Methods

### 2.1. Preparation of the Microcrystallized Borosilicate Bioactive Glass

A borosilicate bioactive glass (BG; 18SiO_2_–6Na_2_O–8K_2_O–8MgO–22CaO–2P_2_O_5_–36B_2_O_3_ (mol%)) was prepared using the melting method [29]. All raw materials, including SiO_2_, Na_2_CO_3_, K_2_CO_3_, (MgCO_3_)_4_·Mg(OH)_2_·5H_2_O, CaCO_3_, NH_4_H_2_PO_4_, and H_3_BO_3_, were of chemical grade and procured from the Sinopharm Chemical Reagent Co., Ltd. (Shanghai, China). The total weight of the raw materials was fixed at 50 g, and according to the composition, the weight of each raw material required was accurately calculated. All raw materials were weighed using an electronic balance (FA1204B, Shanghai Jingketianmei Scientific Instrument Co., Ltd., Shanghai, China) and then mixed and ground in a ceramic mortar. The mixture was placed in a platinum crucible and heated at 1150 °C for 1 h in a high-temperature resistance furnace (YFX12/13Q-YC, Shanghai Yifeng Hot Plates Co., Ltd., Shanghai, China). The melt was quickly poured on a cold stainless-steel plate to quench at room temperature; the resultant glass was smashed into blocks. 

The glass blocks were heated using a corundum crucible at different temperatures for 1 h in a chamber resistance furnace (MXX1100-30, Shanghai Weixing Furnace Industry Co., Ltd., Shanghai, China) and cooled at room temperature. Photographs of BG-600, BG-620, and BG-640 were taken with black cardstock as the background and with blockage of unnecessary light. After being crushed and sieved, glass powders that passed through a 75-mesh sieve but were trapped in a 100-mesh sieve (75-/100-mesh powder, 150–200 µm) and passed through a 200 mesh sieve (200-mesh powder, 75 µm) were obtained. These were named according to the heating temperatures (Table 1). 

### 2.2. Sample Characterization

Differential scanning calorimetry (DSC; 404F3, Netzsch Co., Selb, Germany) was conducted at a heating rate of 10 °C/min from 100 °C to 900 °C to investigate the thermal stability of the BG using the powder samples passed through a 200-mesh sieve. X-ray diffraction (XRD; Smartlab9, Rigaku Co., Tokyo, Japan, α-Cu) analysis was performed to analyze the precipitation of the BG at a scanning rate of 2°/min and a scanning range of 2*θ* = 10°–90°. The morphology and lattice phase of the samples were characterized using high-resolution transmission electron microscopy (HRTEM; JEM-2100F, JEOL Co., Tokyo, Japan).

### 2.3. In Vitro Degradation and Mineralization

Simulated body fluid (SBF) [30] was used to simulate the environment of the human body and test the in vitro degradation and mineralization properties. Its composition is presented in Table 2.

#### 2.3.1. Degradation

In the degradation experiments, the prepared powder samples were immersed in SBF at a solid–liquid ratio of 20 mg:1 mL. In vitro studies were performed under constant temperature and humidity (37 °C and 94% relative humidity). The pH values were determined during the first seven days of immersion without changing the solution. Under the same conditions, samples of the SBF soaking solution were taken on days 1, 3, and 5, and the SBF was changed. The concentrations of B, Si, Ca, Mg, and K in the soaking solution were analyzed via inductively coupled plasma-atomic emission spectroscopy (ICP-AES, Optima 2100DV, Perkin Elmer, Fremont, CA, USA). Moreover, the release of Na was studied using a 0.02 M K_2_HPO_4_ solution (Sinopharm Chemical Reagent Co., Ltd., Shanghai, China) instead of the SBF using the same solid–liquid ratio (20 mg:1 mL) and sampling method. The pH of the K_2_HPO_4_ solution was adjusted to 7.45–7.5 using 1 M HCl.

The concentrations measured using ICP-AES were normalized with the contents of the respective elements in the initial BG and soaking solution:

(1) $$\mathrm{Relative}\;\mathrm{Release}\;(\%)=\frac{C_{X,\;\mathrm{test}\;}-C_{X,\;\mathrm{solution}}}{C_{X,\;\mathrm{BG}}}\times 100\%,$$

where C_X, test_ denotes the concentration of element X measured using ICP-AES; C_X, solution_ indicates the concentration of element X in the soaking solution before adding powder samples; and C_X, BG_ represents the concentration of element X, considering that the entire amount of the BG dissolves in deionized water at a solid–liquid ratio of 20 mg:1 mL. All units must be uniform.

#### 2.3.2. Mineralization

To accurately assess the biomineralization activity according to the unifying approach (0.5 cm^2^ of exposed area per mL of SBF solution) [31], the powder samples passed through a 200-mesh sieve (200-mesh powder, 75 µm) were immersed in the SBF at a solid–liquid ratio of 1.875 mg:1 mL. The samples soaked for 14 d were characterized to evaluate their mineralization property. They were washed with deionized water and dried, and their surface morphologies were observed using field-emission scanning electron microscopy (FESEM; Quanta200FEG, FEI Co., Hillsboro, OR, USA). Secondary electron images were recorded. The accelerating voltage was 10 kV and the working distance was approximately 5 mm from the samples. The Ca/P ratio was determined using energy dispersive spectroscopy (EDS; Apollo X-SDD Det, EDAX Co., Pleasanton, CA, USA) with a resolution of 132 eV.

### 2.4. Cell Proliferation and Cell Viability

#### 2.4.1. Cell Culture

Mouse embryonic osteoblast (OB) precursor cells (MC3T3-E1, iCell Bioscience Inc., Shanghai, China) were incubated in a complete cell medium prepared with fetal bovine serum (10%), a basic medium (90%), and additional dual antibodies (1%). Rat bone marrow mesenchymal stem cells (rBMSCs, School of Medicine, Tongji University, Shanghai, China) were incubated with Dulbecco’s Modified Eagle Medium (DMEM), a low-glucose medium containing fetal bovine serum and dual antibodies. Experiments were performed using fourth-to-seventh-generation rBMSCs. All cell cultures were performed at 37 °C with 5% CO_2_. 

#### 2.4.2. Material Extracts

BG, BG-640, and BG-700 (75-/100-mesh powder, 40 mg) were placed in 40 mL phosphate-buffered saline (PBS, Shanghai Titan Scientific Co., Ltd., Shanghai, China) and maintained in a humidity chamber (37 °C and 94% relative humidity) for 1 d. The supernatant liquid was obtained as a material extract. All three extracts were filtered through a sterilized 0.22 µm filter and stored at 4 °C.

#### 2.4.3. Cell Proliferation and Viability Assay

Cell proliferation and viability were evaluated using the cell-counting kit-8 (CCK-8) method [32,33]. In the cell experiments of the effect of different environmental pH values on cell proliferation, the cell suspensions, including both the OB cells and rBMSCs, were inoculated into 96-well culture plates at a density of 1 × 10^5^ cells/well; the medium was removed after incubation for 24 h. A 100 µL/well DMEM low-glucose medium with different pH values (pH 6, 7, 7.45 (control), 8, and 9) was added for incubation. The samples incubated in the medium with pH = 7.45 were considered the control group. On days 1, 3, and 5, the medium was removed and the 100 µL/well serum-free DMEM low-glucose medium containing 10% CCK-8 was added. After 2 h in the incubator, the optical density (OD) was measured at 450 nm using a microplate reader (Bio-Rad 680, Bio-Rad Laboratories, Inc., Hercules, CA, USA). All the ODs be used for final analyses were obtained by subtracting the OD of a blank well. The cell viability was assessed using LIVE/DEAD staining. Cell samples cultured under different pH environments for 3 d were selected. The staining results were obtained using a confocal fluorescence microscope (SP8; Leica, Wetzlar, Germany) after incubation in the dark for 15 min at room temperature.

In the cell experiment investigating the influence of material extracts on cell proliferation, the cell suspension was inoculated into 96-well culture plates at a density of 5 × 10^3^ cells/well. The control samples (DMEM) and three material extracts were added for incubation at a density of 100 µL/well. The samples without any extracts were set as the control group. The subsequent processes were the same as those discussed earlier.

#### 2.4.4. Statistical Analysis 

All statistical analyses were performed using GraphPad Prism 8.4.3. All experiments were repeated three times (*n* = 3), and the data were presented as mean ± standard deviation. The results were considered statistically significant where *p* < 0.05.

## 3. Results and Discussion

### 3.1. Microcrystallization

The key to the preparation of microcrystallized glasses is the determination of the nucleation and crystallization temperatures. To this aim, DSC measurements were performed to first explore the effect of temperature on the crystallization of the BG and then to determine the optimal heating temperature for microcrystallization. In this section, extrapolated onsets are used for the analyses of the glass transition temperature (T_g_) and the temperature at which crystallization started (T_x_).

**Figure 1:**
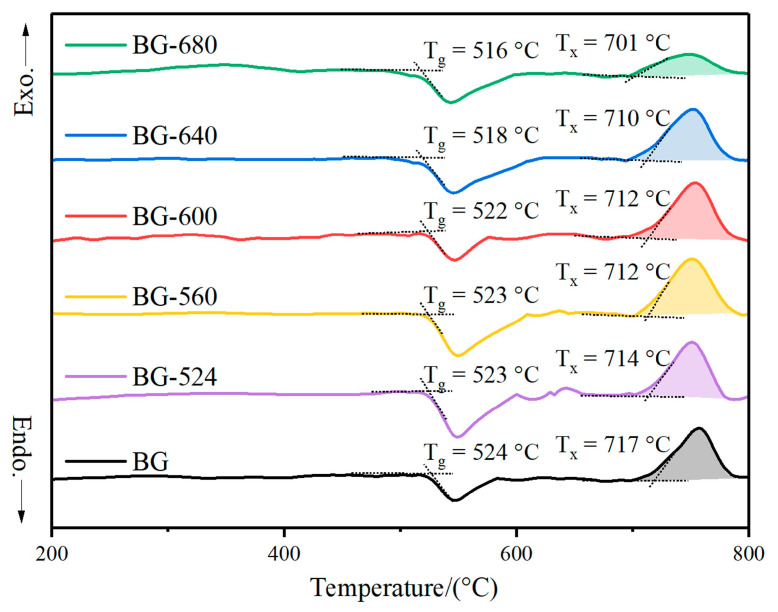
DSC curves of heat-treated BGs (the heating rate was 10 °C/min).

The DSC curve of the original BG without crystallization is presented at the bottom of Figure 1. The T_g_ of the BG was 524 °C, with T_x_ being 701 °C. Usually, the nucleation temperature is >T_g_, while the crystallization temperature is near T_x_. Considering that the nucleation and crystallization temperatures of the BG were close to each other, one-step heat treatment was adopted for microcrystallization.

Accordingly, 524 °C, 560 °C, 600 °C, 640 °C, and 680 °C were tentatively selected as heating temperatures for the DSC analyses (Figure 1). With the increase in heat-treatment temperatures from 524 °C to 600 °C, T_g_ and T_x_ showed a slight decreasing trend. The glassy phase still accounted for the main part of the BG. The heat treatment results in the relaxation of the structure, enhancing its stability. At temperatures beyond 600 °C, T_g_ and T_x_ clearly declined, suggesting that the glassy phase of the BG changed into a microcrystalline phase. The remarkable variations in the structure imply a decline in chemical stability and faster degradation.

Moreover, the areas of the exothermic peaks of crystallization slightly increased from the BG toward the sample heat-treated at 600 °C and showed a decreasing trend for the samples treated at temperatures from 600 °C to 680 °C, especially for BG-680. The increasing trend resulted from the nucleation of the BG because of heat treatment, enhancing the devitrification tendency. Meanwhile, the decrease in the peak area indicates the more extensive devitrification degrees of the glass, decreasing the ability of microcrystals to precipitate again during the DSC analyses. 

**Figure 2:**
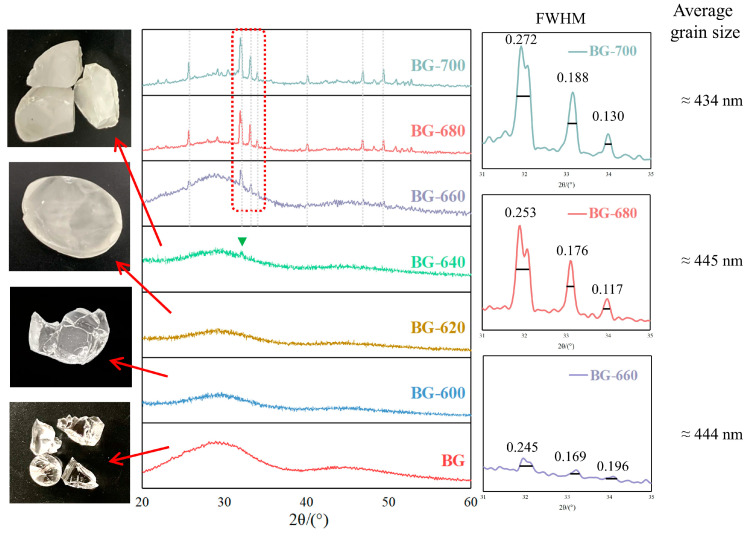
Microcrystallized BGs prepared at different temperatures and the corresponding XRD patterns.

Next, BGs heat-treated at different temperatures from 600 °C to 700 °C at intervals of 20 °C were subjected to XRD analysis (Figure 2). In the XRD pattern of BG-640, a very small precipitation peak is observed. With the increasing heating temperature, the precipitation peaks become clearer in the XRD pattern of BG-660 and are observed as sharp, strong crystal diffraction peaks in the XRD patterns of BG-680 and BG-700. The increase in the intensity of the precipitation peaks indicates increasing crystallinity. Based on the Scherrer equation, the grain size was calculated to be 435–445 nm using the full width at half maxima of the three primary peaks for the samples heat-treated at 660–700 °C. This indicates that increasing the temperature exerted a negligible influence on the grain size for a constant annealing time. Instead, this primarily increased the volume of the microcrystalline phase in the glass.

**Figure 3:**
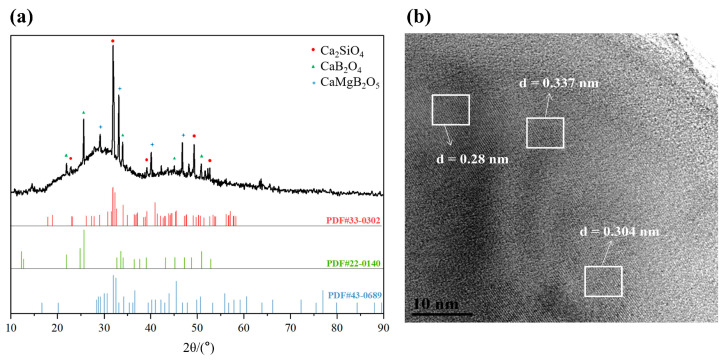
(**a**) XRD analysis of a BG heated at 640 °C for 15 h and (**b**) HRTEM micrograph of BG-640.

As shown in Figure 2, the peaks appear at consistent 2*θ* values irrespective of the temperature. Therefore, the heating temperature did not affect the type of the crystalline phase. To determine the type of the crystalline phase, a BG was heat-treated at 640 °C for 15 h to ensure that the precipitation was as complete as possible. As shown in Figure 3a, the corresponding XRD pattern is consistent with the PDF standard cards of Ca_2_SiO_4_ (PDF#33-0302), CaB_2_O_4_ (PDF#22-0140), and CaMgB_2_O_5_ (PDF#43-0689). In the HRTEM micrographs of BG-640 shown in Figure 3b, the high-resolution image of the dark-contrast region shows clear lattice streaks with spacings of 0.28, 0.337, and 0.304 nm, which correspond to the crystalline phases of CaB_2_O_4_, Ca_2_SiO_4_, and CaMgB_2_O_5_, respectively. According to the TEM and XRD analyses, the main crystalline phase precipitated in the BG was Ca_2_SiO_4_, while CaB_2_O_4_ and CaMgB_2_O_5_ were the secondary crystalline phases.

The photographs shown in Figure 2 reveal that the transparency of the samples decreased upon heating. Thus, the clarified and transparent appearance of BG and BG-600 became milky white and translucent for BG-620, and even less transparent for BG-640. This suggests the presence of microcrystalline phases in BG-620. However, this could not be confirmed via the XRD and HRTEM analyses because of the small quantity and particle size of the crystalline phase. Based on the DSC, XRD, and HRTEM analyses and the appearance of the samples, the desired microcrystalline phases were obtained by heating BG at 620–640 °C, whereas higher heat-treatment temperatures lead to large amounts of microcrystal precipitation.

### 3.2. In Vitro Degradation

When BG is immersed in the SBF, its raw components—CaO, Na_2_O, B_2_O_3_, and SiO_2_—degrade and convert into bioactive constituents, such as Ca^2+^, Na^+^, BO_3_^3−^, and SiO_4_^4−^ [12]. The in vitro degradation of the glass can be evaluated by detecting the concentration of the corresponding bioactive ions. Glass-modifier ions are more susceptible to degradation than network formers. The glass-modifier ion Na^+^ exhibits stronger alkalinity than B(OH)_3_ and Si(OH)_4_ stemming from network formers. Meanwhile, large amounts of Ca^2+^ also increase the alkalinity of the SBF. Therefore, the pH value can also reflect the release process of the bioactive elements in BG.

**Figure 4:**
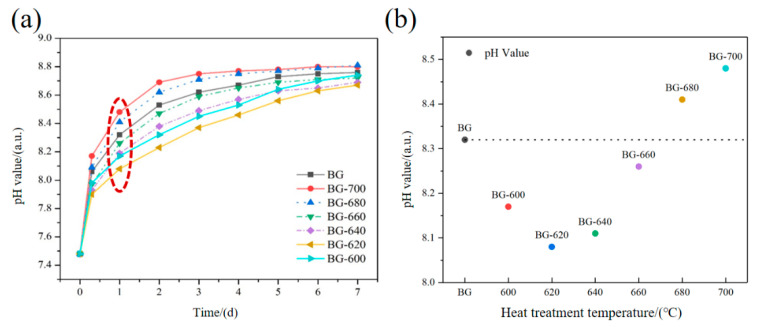
(**a**) pH values of microcrystallized bioactive borosilicate glass (BG) samples immersed in a simulated body fluid for 7 d and (**b**) relationship between the pH value after 1 d of immersion and the heating temperature.

Figure 4a depicts the pH value of the SBF during in vitro degradation. For the BG, the pH value increased with increasing immersion time, with the pH change primarily concentrated in the first and second days. This is because the reaction between the BG and SBF occurred from the surface toward the inside; large quantities of cations (such as Ca^2+^ and Na^+^) in the surface area rapidly elute, resulting in the simultaneous occurrence of a sudden release at the initial stage. Subsequently, the formation of Si-rich barrier layers hinders the erosion of the inner glass by the SBF, resulting in a considerable reduction in the bioactive ingredient supply in the middle and later stages.

After microcrystallization, the pH values showed an overall increasing trend, but the rate of increase was different from that of the BG. Among the heat-treated samples, BG-620 and BG-640 showed lower initial pH growth rates and steady pH growth rates in the later stage, exhibiting a more balanced, near-linear degradation throughout the process. In contrast, BG-680 and BG-700 showed a more rapid initial pH increase and a small pH change at the later stage. This stronger initial abrupt release—compared with that of the BG—is not conducive to degradation regulation.

To further clarify the relationship between the heating temperature and the initial degradation, the pH changes of the different heat-treated samples were compared on day 1. As shown in Figure 4b, the initial pH increase of the SBF was considerably reduced at heating temperatures of 620–640 °C. The heat treatment had a substantial inhibitory effect on the initial burst; however, when the temperature was greater than or equal to 660 °C, the pH of the soaking solution rapidly increased, changing the effect of temperature on the initial degradation of the BG from inhibition to promotion.

These results suggest that an appropriate amount of the microcrystalline phase in the BG is conducive to stabilizing the microstructure of the glass and enhancing its chemical stability, leading to the inhibition of initial degradation. Moreover, devitrification with the formation of the crystalline phases of CaB_2_O_4_, Ca_2_SiO_4_, and CaMgB_2_O_5_ signifies that the overall initial BG composition is gradually depleted of the elements involved during the formation of these crystalline phases. The remaining glassy phase exhibits lower chemical stability compared to that of the initial BG composition. Therefore, excessive microcrystalline phases in the glass accelerate the degradation, which may not be conducive to the inhibition of the initial release and degradation regulation.

### 3.3. Cell Proliferation and Viability

**Figure 5:**
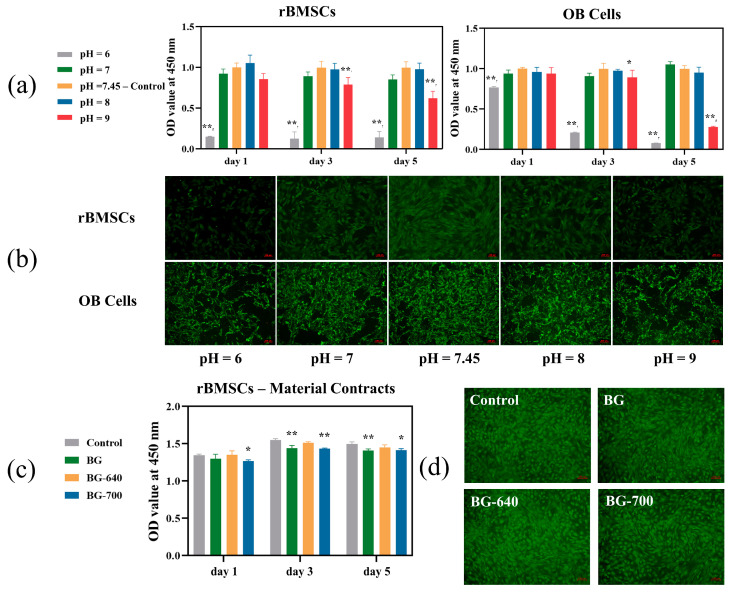
(**a**) Analyses of proliferation activity under different pH conditions: rBMSCs and OB cells (n = 3, *: *p* < 0.05, **: *p* < 0.01, compared to the control group (pH = 7.45)). (**b**) Live/dead fluorescence staining images after incubation for 3 d: rBMSCs and OB cells. (**c**) Analyses of proliferation activity of rBMSCs cultured with material extracts (n = 3, *: *p* < 0.05, **: *p* < 0.01, compared to the control group (rBMSCs cultured without material extracts)). (**d**) Live/dead fluorescence staining images after incubation for 3 d with material extracts.

The environmental pH of human tissues tends to be within a constant range to ensure the proliferative activity of cells. For bone repair, rBMSCs and OB cells are sensitive to the acidic/alkaline environments surrounding bone tissues. We evaluated the effect of different pH levels on the proliferative activity of these two types of cells in vitro using the CCK-8 method. Figure 5a shows that the optimal survival pH of the rBMSCs and OB cells was between 7 and 8. When the ambient pH was <7 or >8, both types of cells exhibited apoptosis to different degrees. The cellular activity further decreased with increasing time. Meanwhile, compared with the OB cells, the rBMSCs showed a more pronounced decrease in activity and higher sensitivity at higher and lower pH values. This was confirmed by the stained fluorescence images presented in Figure 5b. In particular, at pH values between 7 and 8, both types of cells had high numbers, complete morphologies, and good extension. In contrast, at a pH of <7 or >8, the fluorescence highlights in the field of view were clearly reduced, indicating a decrease in cell activity. Therefore, the initial degradation must be controlled to avoid a considerable pH increase.

To further detect the biosafety of the microcrystallized BGs, the material extracts were added to the rBMSCs for cell proliferation and viability assay. Figure 5c shows that although the cellular activity of the rBMSCs with the extracts slightly decreased over time compared with that of the control group, their normal proliferation was relatively stable. In particular, for BG-640, its proliferative activity was almost equivalent to that of the control group. Thus, BGs with various degrees of microcrystallization are biosafe. Moreover, BG-640 exhibited better cell proliferation than the BG, whereas the cell proliferation of BG-700 was slightly worse on the first day compared to that of the BG and was close to it after culturing for 3 and 5 d. This suggests that BGs with different degrees of microcrystallization can influence the activity of rBMSCs to a certain extent by regulating the degradation rate and environmental pH. Figure 5d displays that the fluorescence highlights of BG-640 were slightly brighter than those of the BG and BG-700. This proves that for the BG, a heat treatment at 600–640 °C enhances the cellular activity of the material and ensures biosafety.

### 3.4. Controllable Release of Bioactive Elements

**Figure 6:**
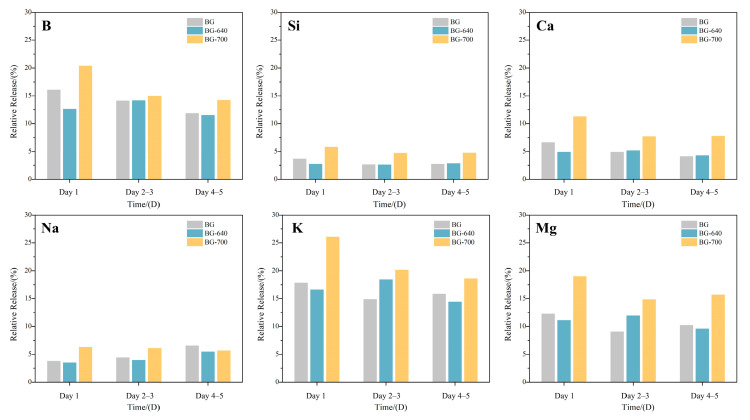
Relative release of bioactive elements from the BG, BG-640, and BG-700 (soaking solution used: SBF for B, Si, Ca, K, and Mg; 0.02 M K_2_HPO_4_ for Na).

Based on the inhibitory/promotional effects of the microcrystallized BGs treated with different temperatures on the pH rise and their biosafety, we further investigated the release of bioactive ingredients at different stages (Figure 6).

By comparing all the elements together, for the BG, whether it was microcrystallized or not, the releases of bioactive elements primarily included BO_3_^3−^, Mg^2+^, K^+^, and Ca^2+^. As the chemical stability of the network former [SiO_4_] is remarkably higher than that of [BO_3_] and [BO_4_], the amount of dissolved Si was lower. The relative release of Ca^2+^ appeared to be lower than that of Mg^2+^ and K^+^, which may have resulted from the high content of CaO in the BG, thereby enlarging the denominator in Equation (1). Notably, the high concentration of Ca^2+^ in the tested soaking solution ceased the dissolution of Ca^2+^. Moreover, Na^+^ was tested using a 0.02 M K_2_HPO_4_ solution to avoid interference from Na^+^ in the SBF; thus, its comparison was not appropriate.

Thereafter, for the non-microcrystallized BG sample, large amounts of bioactive elements were detected in the soaking solution on the first day. Alkali and alkaline earth metal ions such as Na^+^, K^+^, Mg^2+^, and Ca^2+^ were still abundant in the surface area at the beginning. Thus, ion exchange with H^+^ was less hindered. Furthermore, surface erosion was easier for the glass network formers. Thus, the initial release of all the bioactive elements was large. With time, the release of bioactive elements decreased. The inward degradation contributed to ion deficiency at the surface as well as ion migration and dissolution difficulties. However, at the later stage, unlike B, Si, and Ca, elemental release, including Na, Mg, and K, showed a decline followed by a gradual increase, especially for Na. This can be attributed to the reduction in the stability of the remaining glass structure after erosion, promoting dissolution. Therefore, the ICP analyses reconfirm that the bioactive elements were released rapidly at the initial stage but insufficiently at the later stage.

For the BG-640 sample, the release of B, Si, Ca, and Mg substantially decreased, by 21.58%, 26.51%, 16.53%, and 9.53%, respectively, compared with that of the BG after the first day. These elements are immobilized in the form of crystals, effectively blocking the erosion of the SBF. At the same time, heat-preservation treatment at relatively low temperatures also facilitates the release of stress in glass, so that the stability is improved. This is the reason why cations (including K^+^ and Na^+^) declined by 6.77% and 7.15%, respectively. During the latter 4 d of degradation, the release of each bioactive element still showed a gradual decreasing trend, but the release of B, Si, Ca, and Na was close to that of the BG, ensuring sustainable release during the later stage. Notably, the release from the BG-640 surpassed that of the BG. This may have been influenced by the precipitation of the surrounding microcrystals containing B. Cations not involved in microcrystal formation migrate and dissolve readily, which can be expected to have a positive effect on the mineralization of the BG. Therefore, in terms of the overall degradation of BG-640, sudden releases of bioactive elements at the initial stage can be controlled via appropriate amounts of microcrystals. The equilibrium of the entire degradation process is improved.

During the entire test period, the release of the tested elements for BG-700 was far higher than that of the BG and BG-640, especially on the first day. This is crucial for the positive role in in vitro mineralization. However, the release of all the tested elements encounters the same problem—an abrupt reduction in ion release. As discussed in Section 3.2, copious amounts of microcrystalline phase weaken the chemical stability of the remaining amorphous phase, resulting in a faster release of bioactive elements and the hindered equilibrium of the whole degradation.

Overall, from the whole degradation of BG-640 and BG-700, the release of bioactive components is controllable by varying the amounts of microcrystals in the glass. A low amount of the crystalline phase improves the equilibrium of degradation, while too much of the crystalline phase is counterproductive.

### 3.5. Mineralization Properties

The mineralization process of the BG involves the precipitation of Ca^2+^, the dissolution of the glass network structure, the formation of Si-rich layers, the migration of Ca^2+^ to the surface, and the combination of Ca^2+^ with PO_4_^3−^ to form Ca–P deposits that lead to crystallization [34]. These Ca–P layers crystallize to produce hydroxyapatite (HAP), which is required for human bone growth.

**Figure 7:**
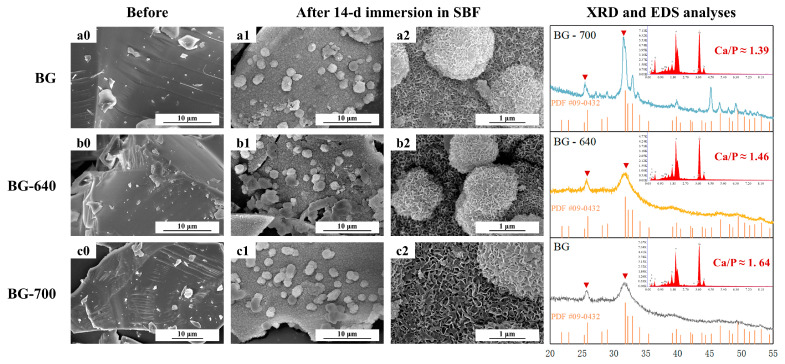
SEM micrographs of the BG, BG-640, and BG-700 with/without immersion in an SBF: (**a0**,**b0**,**c0**) BG, BG-640 and BG-700, before immersion; (**a1**,**a2**) BG, after 14-d immersion in SBF; (**b1**,**b2**) BG-640, after 14-d immersion in SBF; (**c1**,**c2**) BG-700, after 14-d immersion in SBF. XRD and EDS analyses of the BG, BG-640, and BG-700 after immersion in the SBF for 14 d are listed on the right side.

The in vitro mineralization of the BG, BG-640, and BG-700 powder samples immersed for 14 d was observed via FESEM (Figure 7). Figure 7(a0), Figure 7(b0) and Figure 7(c0), respectively, show that the surfaces of all the samples before immersion were smooth and flat, with a few broken residual glass particles attached and some scratches because of mechanical disruption. In contrast, after soaking for 14 d (Figure 7(a1), Figure 7(b1) and Figure 7(c1), respectively), the surfaces of the samples became coarse and porous. Consequently, certain spherical particles appeared. Notably, a greater number of spherical particles were present on the surfaces of BG-640 and BG-700 compared to the BG surface, indicating improved mineralization to a certain degree. Figure 7(a2), Figure 7(b2) and Figure 7(c2), respectively, display more detailed micrographs of the products. All of the samples displayed the same loose deposits with nanostructured pores on their surfaces. It seems like the surfaces and spherical particles comprised the accumulation of tiny particles in flakes or bars, indicating the growth of mineralization products.

To determine whether the products were HAP, we performed XRD and EDS analyses. According to the XRD patterns, although the intensities of peaks of the BG and BG-640 were not extremely strong, the characteristic peaks adequately fitted the PDF #09-0432. Owing to the microcrystalline phase for BG-700, clear detection of the product types was difficult. However, the two peaks portrayed in Figure 7 tended to split, and the new tiny peaks fitted PDF #09-0432 well. This proved that the mineralization products were Ca_5_(PO_4_)_3_(OH) (HAP, PDF #09-0432). 

Based on the EDS analyses, the ratios of Ca to P (Ca/P) of the products on the surfaces of the BG, BG-640, and BG-700 were 1.39, 1.46, and 1.64 respectively. Compared with Ca/P = 1.67 (the ratio of Ca to P of HAP), the products of BG-700 were the closest to HAP. These results show that microcrystalline phases can also guide the growth of HAP, which is conducive to mineralization.

## 4. Conclusions

The microcrystallization of BGs has a remarkable effect on the stability of their microstructure, the release of bioactive elements, and in vitro degradation and mineralization. Under an optimal heat treatment at 620–640 °C for 1 h, appropriate amounts of microcrystalline phases in the glasses can be conducive to weakening the initial release of bioactive elements, including glass network formers (B and Si) and glass-modifier cations (Na^+^, K^+^, Mg^2+^, and Ca^2+^), whereas excessive crystallization produced under higher temperatures accelerates it. The degrees of microcrystallization can influence the equilibrium of the whole degradation and control the release of bioactive elements. Moreover, microcrystallized BGs proved to be biosafe. The decreases in the pH rise and ion release protect pH-sensitive rBMSCs and OB cells to some extent. The gentle release of bioactive elements at the initial stage provides a sustainable release of bioactive elements at the later stage, improving the in vitro mineralization ability. Meanwhile, microcrystalline phases proved to be conducive to the growth of HAP. This work may provide a new approach to developing bone-repair materials to achieve controllable degradation and better bioactivity.

## Figures and Tables

**Table 1:** Processing protocols of the investigated borosilicate bioactive glass (BG).

Glass	BG-524	BG-560	BG-600	BG-620	BG-640	BG-660	BG-680	BG-700
Melting (°C)	1150							
Melting time (h)	1							
Heating (°C)	524	560	600	620	640	660	680	700
Heating time (h)	1							

**Table 2:** Composition of SBF.

Ion	Na^+^	K^+^	Mg^2+^	Ca^2+^	Cl^−^	HCO_3_^−^	HPO_4_^2−^	SO_4_^2−^	pH
Concentration (mmol·L^−1^)	142	5	1.5	2.5	147.8	4.2	1	0.5	7.45–7.5

## Data Availability

Data are contained within the article.

## References

[1] Dukle A., Murugan D., Nathanael A.J., Rangasamy L., Oh T.H. (2022). Can 3D-Printed Bioactive Glasses Be the Future of Bone Tissue Engineering?. Polymers.

[2] Ren X.X., Chen X., Geng Z., Su J.C. (2022). Bone-targeted biomaterials: Strategies and applications. Chem. Eng. J..

[3] Xia Y.L., Wang H.Y., Li Y.H., Fu C.F. (2022). Engineered bone cement trigger bone defect regeneration. Front. Mater..

[4] Fernandes H.R., Gaddam A., Rebelo A., Brazete D., Stan G.E., Ferreira J.M.F. (2018). Bioactive Glasses and Glass-Ceramics for Healthcare Applications in Bone Regeneration and Tissue Engineering. Materials.

[5] Yao A.H., Wang D.P., Fu Q., Huang W.H., Rahaman M.N. (2007). Preparation of bioactive glasses with controllable degradation behaviour and their bioactive characterization. Chin. Sci. Bull..

[6] Schuhladen K., Pantulap U., Engel K., Jelen P., Olejniczak Z., Hupa L., Sitarz M., Boccaccini A.R. (2021). Influence of the replacement of silica by boron trioxide on the properties of bioactive glass scaffolds. Int. J. Appl. Glass Sci..

[7] Fu Q.A., Rahaman M.N., Bal B.S., Bonewald L.F., Kuroki K., Brown R.F. (2010). Silicate, borosilicate, and borate bioactive glass scaffolds with controllable degradation rate for bone tissue engineering applications. II. In vitro and in vivo biological evaluation. J. Biomed. Mater. Res. A.

[8] Hench L.L. (2006). The story of Bioglass (R). J. Mater. Sci.-Mater. M.

[9] Hench L.L. (1998). Bioactive materials: The potential for tissue regeneration. J. Biomed. Mater. Res..

[10] Kaur G., Pandey O.P., Singh K., Homa D., Scott B., Pickrell G. (2014). A review of bioactive glasses: Their structure, properties, fabrication, and apatite formation. J. Biomed. Mater. Res. A.

[11] Day R.M. (2005). Bioactive glass stimulates the secretion of angiogenic growth factors and angiogenesis in vitro. Tissue Eng..

[12] Huang W.H., Day D.E., Kittiratanapiboon K., Rahaman M.N. (2006). Kinetics and mechanisms of the conversion of silicate (45S5), borate, and borosilicate glasses to hydroxyapatite in dilute phosphate solutions. J. Mater. Sci.-Mater. M.

[13] Uysal T., Ustdal A., Sonmez M.F., Ozturk F. (2009). Stimulation of Bone Formation by Dietary Boron in an Orthopedically Expanded Suture in Rabbits. Angle Orthod..

[14] Vitale-Brovarone C., Miola M., Balagna C., Verne E. (2008). 3D-glass-ceramic scaffolds with antibacterial properties for bone grafting. Chem. Eng. J..

[15] Liu X., Huang W.H., Fu H.L., Yao A.H., Wang D.P., Pan H.B., Lu W.W. (2009). Bioactive borosilicate glass scaffolds: Improvement on the strength of glass-based scaffolds for tissue engineering. J. Mater. Sci.-Mater. M.

[16] Shafaghi R., Rodriguez O., Wren A.W., Chiu L., Schemitsch E.H., Zalzal P., Waldman S.D., Papini M., Towler M.R. (2021). In vitro evaluation of novel titania-containing borate bioactive glass scaffolds. J. Biomed. Mater. Res. A.

[17] Hamadouche M., Meunier A., Greenspan D.C., Blanchat C., Zhong J.P.P., La Torre G.P., Sedel L. (2001). Long-term in vivo bioactivity and degradability of bulk sol-gel bioactive glasses. J. Biomed. Mater. Res..

[18] Lin S., Van den Bergh W., Baker S., Jones J.R. (2011). Protein interactions with nanoporous sol-gel derived bioactive glasses. Acta Biomater..

[19] Gaddam A., Golebiewski P., Fernandes H.R., Pysz D., Neto A.S., Diduszko R., Malinowska A., Stepien R., Cimek J., Buczynski R. (2021). Development of microfibers for bone regeneration based on alkali-free bioactive glasses doped with boron oxide. J. Am. Ceram. Soc..

[20] Ning J., Yao A., Wang D.P., Huang W.H., Fu H.L., Liu X., Jiang X.Q., Zhang X.L. (2007). Synthesis and in vitro bioactivity of a borate-based bioglass. Mater. Lett..

[21] Fu L., Engqvist H., Xia W. (2020). Glass-Ceramics in Dentistry: A Review. Materials.

[22] Xiao H., Peng W., Deng C. (2000). Preparation technology, properties and applications of microcrystalline ceramics. China Ceram..

[23] Chen X.J., Chen X.H., Brauer D.S., Wilson R.M., Hill R.G., Karpukhina N. (2014). Bioactivity of Sodium Free Fluoride Containing Glasses and Glass-Ceramics. Materials.

[24] Chen Q.Z., Liang S.L., Wang J., Simon G.P. (2011). Manipulation of mechanical compliance of elastomeric PGS by incorporation of halloysite nanotubes for soft tissue engineering applications. J. Mech. Behav. Biomed..

[25] Chen Q.Z., Boccaccini A.R. (2006). Coupling mechanical competence and bioresorbability in Bioglass (R)-derived tissue engineering scaffolds. Adv. Eng. Mater..

[26] Chen C., Ding J.X., Wang H., Wang D.P. (2022). Nd-doped Mesoporous Borosilicate Bioactive Glass-ceramic Bone Cement. J. Inorg. Mater..

[27] Lu L.N., Lin J., Gu Y.F., Zhang X., Zhao Y.S., Huang W.H. (2012). Effect of the crystallization treatment of bioactive borate-based glass containing strontium on its degradation and bio-activity in vitro. J. Funct. Mater..

[28] Madshal M.A., Abdelghany A.M., Abdelghany M.I., El-Damrawi G. (2022). Biocompatible borate glasses doped with Gd_2_O_3_ for biomedical applications. Eur. Phys. J. Plus.

[29] Yao A.H., Wang D.P., Huang W.H., Fu Q., Rahaman M.N., Day D.E. (2007). In vitro bioactive characteristics of borate-based glasses with controllable degradation behavior. J. Am. Ceram. Soc..

[30] Kokubo T., Kushitani H., Sakka S., Kitsugi T., Yamamuro T. (1990). Solutions Able to Reproduce Invivo Surface-Structure Changes in Bioactive Glass-Ceramic A-W3. J. Biomed. Mater. Res..

[31] Popa A.C., Stan G.E., Husanu M.A., Mercioniu I., Santos L.F., Fernandes H.R., Ferreira J.M.F. (2017). Bioglass implant-coating interactions in synthetic physiological fluids with varying degrees of biomimicry. Int. J. Nanomed..

[32] Sun H., Ge W., Gao X., Wang S., Jiang S., Hu Y., Yu M., Hu S. (2015). Effect of SDT on the survival rate of endometrial cancer cells, assessed by the CCK-8 method. PLoS ONE.

[33] Gao J., Bin Y.E., Wei-Hua W.U., Kong J. (2010). Tachyzoites of Toxolasma gondii enhances the cytotoxicity of Etooside (V-16) to mouse colon cancer cell ct26 in vitro. Chin. J. Zoonoses.

[34] Yao A.H., Lin J., Duan X., Huang W.H., Rahaman M.N. (2008). Formation mechanism of multilayered structure on surface of bioactive borosilicate glass. Chin. J. Inorg. Chem..

